# Nutritional Status and Linear Growth at Diagnosis and Their Association with Clinical Outcomes in Paediatric Inflammatory Bowel Disease: A Multicentre Cohort Study

**DOI:** 10.3390/nu18172928

**Published:** 2026-09-07

**Authors:** Chiara Cesari, Carla Maria Grimaldi, Matteo Motta, Viola Carzaniga, Chiara Maria Trovato, Giovanna Zuin, Lorenzo D’Antiga, Naire Sansotta

**Affiliations:** 1 Department of Pediatrics, University of Milan Bicocca, 20900 Monza, Italy; c.cesari2@campus.unimib.it (C.C.); c.grimaldi8@campus.unimib.it (C.M.G.); v.carzaniga@campus.unimb.it (V.C.); ldantiga@asst-pg23.it (L.D.); 2 Department of Pediatric Hepatology, Gastroenterology and Transplantation, Hospital Papa Giovanni XXIII, 24127 Bergamo, Italy; motta.matteo@asst-pg23.it; 3 Digestive Disease and Nutritional Rehabilitation Unit, Bambino Gesù Children Hospital, IRCCS, 00165 Rome, Italy; chiaramariatrovato@gmail.com; 4 Department of Pediatrics, Fondazione IRCCS San Gerardo dei Tintori, 20900 Monza, Italy

**Keywords:** paediatric inflammatory bowel disease, Crohn’s disease, ulcerative colitis, nutritional status, growth impairment, body mass index, disease relapse, biologic therapy, enteral nutrition

## Abstract

**Background/Objectives**: Malnutrition is common in paediatric inflammatory bowel disease (IBD) and may affect growth, disease severity, and clinical outcomes. The aim of our study was to assess nutritional status and linear growth at diagnosis and during follow up and to evaluate their association with clinical outcomes in paediatric IBD. **Methods**: This multicentre retrospective cohort study included children diagnosed with Crohn’s disease (CD) or ulcerative colitis (UC) at two paediatric IBD referral centres. Clinical, biochemical, and anthropometric data were collected at diagnosis and throughout follow-up. Underweight was defined as BMI z-score ≤ −2; growth failure (stunting) was defined as height-for-age z-score ≤ −2. **Results**: Among 128 children with IBD (69 CD, 59 UC; mean age: 157.2 ± 29.5 months), 23% (29/128) were underweight at diagnosis, 68% (87/128) had normal BMI, and 9% (12/128) were overweight at diagnosis. Underweight status was more frequent in the CD than UC groups (30% vs. 15%; *p* = 0.022). Growth failure affected 7.0% of patients at diagnosis and was observed in 5.3% of patients with available data at 36 months. Stunting was more frequent in CD than UC. Biologic therapy was more frequently initiated in underweight patients (41% vs. 17%, *p* = 0.06). At a mean follow-up of 29.4 months, 86% (25/29) of underweight patients achieved a normal or elevated BMI. Underweight status was not associated with an increased risk of relapse (adjusted OR 0.52, 95% CI 0.19–1.30; adjusted HR 0.60, 95% CI 0.28–1.29). **Conclusions**: Malnutrition is frequent at diagnosis in paediatric IBD, particularly in Crohn’s disease, and is associated with increased inflammatory burden. Although most patients showed improvement in nutritional status during follow-up, baseline underweight status was not independently associated with subsequent disease relapse. These findings highlight the importance of early and longitudinal assessment of nutritional status and linear growth as integral components of paediatric IBD management.

## 1. Introduction

Inflammatory bowel diseases (IBD) are relapsing inflammatory disorders of the gastrointestinal tract, with a multifactorial aetiology that remains poorly understood. Onset during childhood typically occurs in approximately 25% of cases [1,2,3,4].

Nutrition has been implicated in all aspects of paediatric IBD: from disease pathogenesis to diagnosis, treatment response, and long-term prognosis [5,6]. Additionally, malnutrition is frequently present at disease presentation and may chronically persist during disease course with critical consequences for body composition, growth, puberal development, bone health, quality of life, and therapeutic outcomes [5,7,8].

Several factors can influence the nutritional status of patients with IBD. Among these, inadequate nutrient intake due to food aversion, as well as nutrient losses resulting from malabsorption, can disrupt nutrient balance and increase the risk of malnutrition [5,6,9,10]. Additionally, the activation of the immune system and the demands of tissue repair machinery may further elevate nutritional requirements due to increased nutrient utilisation and altered metabolic processes [8,10,11].

Malnutrition is more commonly found in patients with Crohn’s disease (CD) compared to those with ulcerative colitis (UC). Up to 60–80% of CD patients are reported to be underweight and have a low BMI at diagnosis, while only 30–40% of children with UC present similar features [11,12]. This difference is mainly explained by the different pathogenetic mechanisms of inflammation in the two conditions. In CD, inflammation is transmural with frequent involvement of the small intestine, the site of nutrient absorption; in UC, inflammation may involve only the mucosa layer and is limited to the last portions of the large intestine [13,14,15]. Nevertheless, children with UC remain susceptible to nutritional deficiency, which may contribute to disease progression and reduced therapeutic response [16].

Given the high prevalence and clinical relevance of altered nutritional status in paediatric IBD, early longitudinal nutritional and growth assessment is crucial. Anthropometric measurements, including weight, height and BMI, should be systematically recorded at diagnosis and throughout follow-up. In clinical practice, BMI remains widely used because of its simplicity, accessibility, and availability for longitudinal assessment, particularly in retrospective multicentre studies [5,17]. Its interpretation should therefore be complemented, whenever possible, by assessment of linear growth and body composition.

Nevertheless, recent data from the ESPGHAN Porto Group highlight substantial variability in nutritional assessment across paediatric IBD centres, with body composition and height velocity still not routinely assessed in clinical practice [18].

Despite the recognised burden of malnutrition in paediatric IBD, longitudinal data integrating nutritional and linear growth trajectories with treatment exposure and subsequent clinical outcomes remain limited. In particular, the prognostic significance of nutritional status at diagnosis and its relationship with disease course have not been fully established. Longitudinal studies are therefore needed to determine whether early nutritional impairment identifies distinct disease trajectories and may contribute to risk stratification in paediatric IBD.

The primary aim of this study was to assess the prevalence of malnutrition at diagnosis in a multicentre cohort of children with IBD.

Secondary aims were to assess longitudinal changes in nutritional status and linear growth, evaluate their relationship with nutritional and medical interventions, and investigate the association between baseline nutritional status and subsequent disease relapse.

## 2. Materials and Methods

This multicentric retrospective cohort study was conducted at two Paediatric IBD Referral Centres (ASST Papa Giovanni XXIII hospital in Bergamo and IRCC San Gerardo Hospital in Monza, Italy).

Children (<18 years) with a diagnosis of UC or CD established according to the Porto criteria were included. Patients with very early-onset IBD (VEO-IBD) or IBD-unclassified (IBD-U), not subsequently reclassified, were excluded to ensure cohort homogeneity.

Clinical data were retrieved from electronic health records and medical charts. Demographic characteristics, disease type, and location were recorded. Anthropometric parameters such as body height [BH], body weight [BW], and body mass index [BMI] were collected at diagnosis and throughout follow-up. Height-for-age and BMI-for-age z-scores were calculated according to the CDC growth references. Linear growth was assessed using height-for-age z-scores (HAZ), with stunting defined as HAZ ≤ −2 SD. Nutritional status was assessed using BMI-for-age z-scores, with underweight defined as a BMI-for-age z-score ≤ −2 SD. HAZ and BMI-for-age z-scores were recorded at diagnosis and at 6-, 12-, 18-, 24-, and 36-month follow-up. Pubertal status at diagnosis, when available, was categorised as prepubertal, pubertal, or post pubertal according to Tanner staging.

Laboratory parameters (erythrocyte sedimentation rate [ESR], C-reactive protein [CRP], haemoglobin, platelet count, serum albumin, ferritin, and faecal calprotectin) and treatment data (immunomodulators, biologics, and nutritional therapy) were collected.

Longitudinal follow-up data were collected at 6-month intervals, spanning from 6 months post-diagnosis to 36 months post-diagnosis. At each time point, clinical, anthropometric, and laboratory data, as well as disease relapse, were assessed.

Disease location was classified according to the Paris classification, and disease activity was evaluated using the Paediatric Crohn’s Disease Activity Index (PCDAI) and Paediatric Ulcerative Colitis Activity Index (PUCAI). Relapse was defined as PCDAI ≥12.5 for CD and PUCAI ≥ 10 for UC, together with treatment escalation (corticosteroids, biologics, or change in maintenance therapy).

Given the retrospective design of the study, no a priori sample size calculation was performed. All consecutive patients meeting the eligibility criteria during the predefined study period were included.

The present retrospective study was conducted using data from the paediatric IBD database, with formal approval from the Ethics Committee (protocol no. 2018-0105; Resolution no. 1089, 14 June 2018). The study was conducted in accordance with the Declaration of Helsinki and applicable Good Clinical Practice principles. Written informed consent for the collection and use of clinical data for research purposes was obtained in accordance with the approved protocol.

Data are presented as counts and percentages for categorical variables, and as means with the standard deviation (SD) or medians with ranges, as appropriate. Groups were compared using the chi-square test for categorical variables and t-test for continuous variables. The Shapiro–Wilk test was used to assess normality. For non-normally distributed variables, the Mann–Whitney U test was applied. Population proportions were compared using the z-test. A *p*-value < 0.05 was considered statistically significant.

The association between underweight status at diagnosis and subsequent relapse was evaluated using the chi-square test, the Kaplan–Meier estimation of relapse-free survival with the log-rank test, the occurrence of relapse with multivariable logistic regression, and time to first relapse with Cox proportional hazards regression. Models were adjusted for disease type, sex, age at diagnosis, and biologic therapy at induction. The results are reported as odds ratios (ORs) and hazard ratios (HRs) with 95% confidence intervals (CIs).

## 3. Results

### 3.1. Malnutrition at Baseline

#### 3.1.1. Cohort Characteristics and Prevalence of Malnutrition

The study cohort included 128 children with IBD (CD 69, UC 59), predominantly Caucasian (63%), followed by African (11%), with a sex distribution M:F of 1.4:1. The mean age at diagnosis was 157.2 months (SD = 29.5; range: 80 to 212 months).

At diagnosis, 29/128 (23%) patients were underweight, including 21 patients with CD and 8 with UC; 87/128 (68%) had a normal BMI; and 12/128 (9%) were overweight. The mean BMI for the age z-score at diagnosis was 18.1 kg/m^2^ (SD = 3.26).

Linear growth was assessed separately using height-for-age z-scores (HAZ). At diagnosis, 9/128 (7%) patients met the criteria for stunting (HAZ ≤ −2 SD). Stunting was observed in 7/69 (10.1%) patients with CD and 2/59 (3.4%) patients with UC. Although more frequent in CD, the difference did not reach statistical significance (*p* = 0.253).

These findings indicate that nutritional impairment at diagnosis was predominantly characterised by underweight rather than linear growth impairment.

Furthermore, pubertal status data were available for 72/128 (56%) patients, including 37 with CD and 35 with UC. Among patients with CD, 10/37 (27%) were prepubertal, 17/37 (46%) pubertal, and 10/37 (27%) post pubertal. In the UC group, 6/35 (17%) were prepubertal, 12/35 (34%) pubertal, and 17/35 (49%) post pubertal. The distribution of pubertal status did not differ significantly between the CD and UC groups (*p* = 0.163).

Baseline demographic and clinical characteristics according to BMI category are summarised in Table 1.

#### 3.1.2. Risk Factors of Malnutrition

Underweight status was more frequent in males (27%, 20/75) than in females (17%, 9/53), although the difference was not statistically significant (*p* = 0.20). No significant differences were observed in age at diagnosis (*p* = 0.175) or diagnostic delay (4.76 ± 4.88 vs. 6.70 ± 7.18 months; *p* = 0.17) between the underweight and normal/overweight groups.

Stratified by disease type, underweight was significantly more common in CD (30%, 21/69) than in UC (15%, 9/59; *p* = 0.022). Ileal involvement was present in 58% of CD patients (40/69), of whom 11/40 (28%) were underweight (*p* = 0.50). Among patients with UC, 35/59 (59%) had pancolitis, of whom 6/35 (17%) were underweight (*p* = 0.60).

At diagnosis, severe disease (S1) was observed in 44% of UC patients, (26/59), but only 8% (2/26) were underweight. In CD patients, 24% (17/69) presented with stricturing or penetrating disease (B2/B3), with a higher proportion of underweight patients (59%; 10/17, *p* = 0.003).

#### 3.1.3. Disease Activity and Laboratory Biomarkers

At baseline, disease activity scores were not significantly associated with nutritional status. In CD patients, the mean PCDAI was 27.69 (SD = 17.8) with no significant difference between underweight and normal/overweight (*p* = 0.9). In UC patients, the mean PUCAI was 39.9 (SD = 24.3) and was lower in underweight patients (mean = 25 SD = 20.2) compared to those with normal/overweight (mean = 42.3; SD = 24; *p* = 0.1).

Laboratory and faecal parameters according to BMI category at diagnosis are reported in Table 2. Notably, underweight patients exhibited significantly higher rates of both elevated ESR (*p* = 0.02) and thrombocytosis (*p* = 0.03) compared with children with normal/overweight counterparts.

#### 3.1.4. Treatment and Nutritional Therapy

At induction, biologic therapy was initiated in 41% (12/29) of underweight patients compared to 17% (17/99) of those with normal/overweight (*p* = 0.06). Most patients who receiving biologics had CD (86%, 25/29).

In the CD subgroup, exclusive enteral nutrition was used as induction therapy in 70% of patients (48/69), of whom 35% (17/48) were underweight (*p* = 0.17).

### 3.2. Longitudinal Changes in Growth and Nutritional Status over Time

#### 3.2.1. BMI Trajectories

Among the 29 patients who were underweight at diagnosis, 86% (*n* = 25) had a normal or elevated BMI at follow-up (normal weight *n* = 23; overweight *n* = 2), while 14% (*n* = 4) remained underweight.

Among the 87 patients with normal BMI at diagnosis, 62% (*n* = 54) remained normal weight, 2% (*n* = 2) became underweight, and 13% (*n* = 11) became overweight. Of the 12 patients who were overweight at diagnosis, 67% (*n* = 8) remained overweight and 33% (*n* = 4) had a normal BMI at follow-up.

Among patients who were underweight at diagnosis, the mean BMI-for-age z-score increased from −2.76 at baseline to −0.66 at the 36-month follow-up.

**Figure 1:**
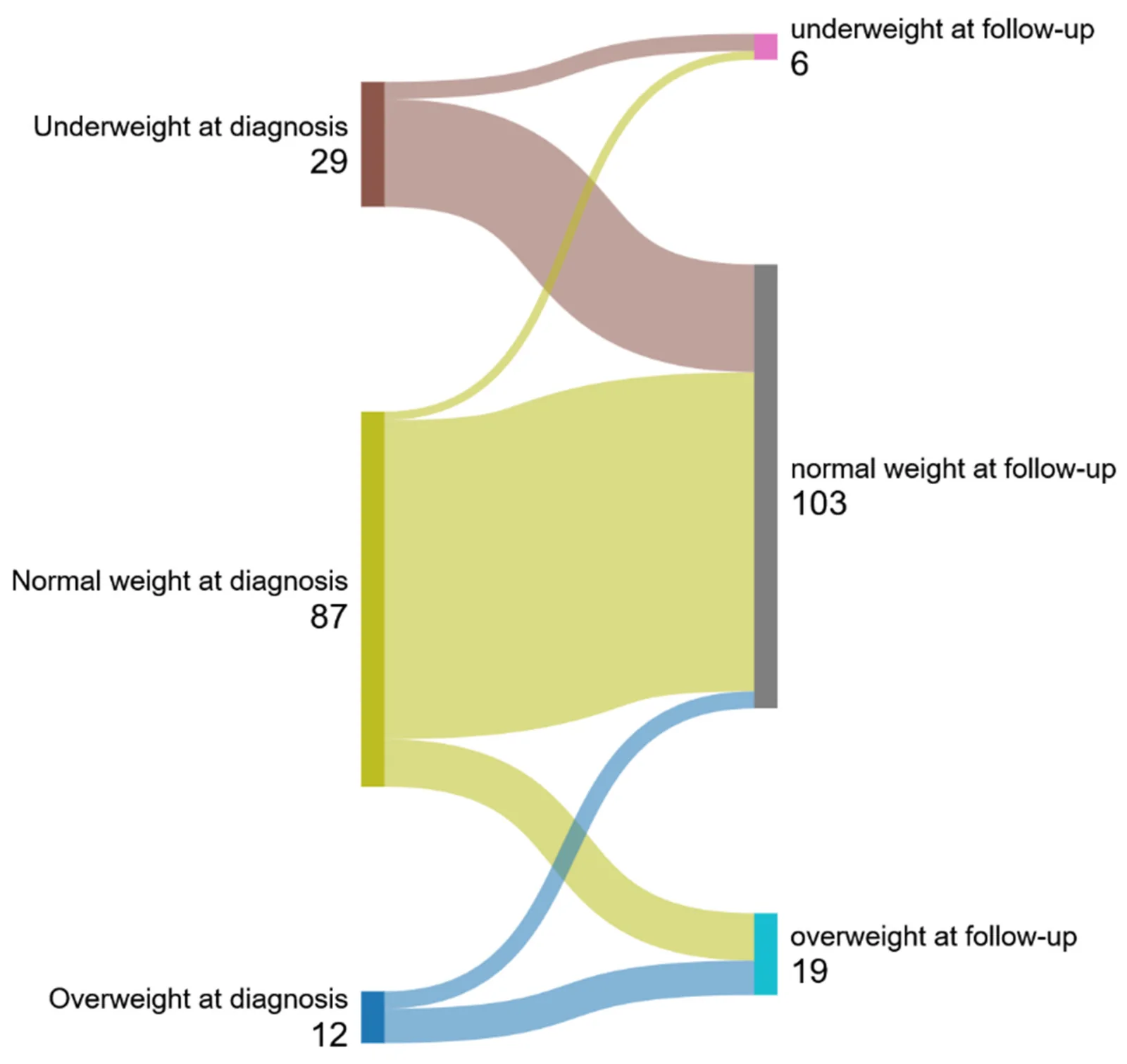
Transitions between BMI categories from diagnosis to follow-up in paediatric inflammatory bowel disease.

Changes in BMI categories over time are illustrated in Figure 1.

The figure shows changes in nutritional status from diagnosis to follow-up. Most patients maintained or achieved normal weight during follow-up, while only a minority remained underweight or developed overweight status.

#### 3.2.2. Longitudinal Changes in Linear Growth

At diagnosis, stunting (HAZ ≤ −2 SD) was present in 9/128 (7.0%) patients. At 36 months, 4/75 (5.3%) patients with available anthropometric data met the criteria for stunting.

Among patients with CD, the prevalence of stunting was 10.1% (7/69) at diagnosis and 6.7% (3/45) at 36 months. In patients with UC, stunting was observed in 3.4% (2/59) at diagnosis and 3.3% (1/30) at 36 months (Table 3).

#### 3.2.3. Nutritional Status in Relation to Nutritional and Therapeutic Interventions

After a mean follow-up period of 29.4 months, most patients who were underweight at diagnosis showed nutritional recovery, with the mean BMI-for-age z-score increasing from −2.76 at diagnosis to −0.66 after 36 months of follow-up. At the final follow-up, among patients receiving biologic therapy, 3/54 (5.6%) were underweight, whereas 51/54 (94.4%) had a normal or elevated BMI; nutritional status at the final follow-up was not significantly associated with biologic therapy (*p* = 0.69).

In the CD subgroup, exclusive enteral nutrition (EEN) was used as induction therapy in 48/69 (70%) patients, of whom 17/48 (35%) were underweight at diagnosis. EEN use did not differ significantly according to baseline nutritional status (*p* = 0.17). At follow-up, only 1/17 (6%) of the initially underweight patients treated with EEN remained underweight. Partial enteral nutrition (PEN) was used as maintenance therapy in 24/69 (35%) patients with CD, including 6 patients who were underweight at diagnosis. At follow-up, 5/6 (83%) of these patients were no longer underweight.

#### 3.2.4. Association Between Baseline Nutritional Status and Subsequent Disease Relapse

After a mean follow-up of 29.4 months, 54/128 (42%) patients experienced at least one disease relapse. In univariate analysis, underweight status at diagnosis was not significantly associated with the risk of relapse (OR 0.44, 95% CI 0.17–1.05; *p* = 0.075). At 36 months, relapse-free survival was 67% in patients who were underweight at diagnosis compared with 49% in those with normal weight or overweight; however, this difference did not reach statistical significance (log-rank *p* = 0.087). In multivariable analysis adjusted for disease type, age, sex, and biologic therapy at induction, underweight status at diagnosis was not independently associated with relapse risk.

**Figure 2:**
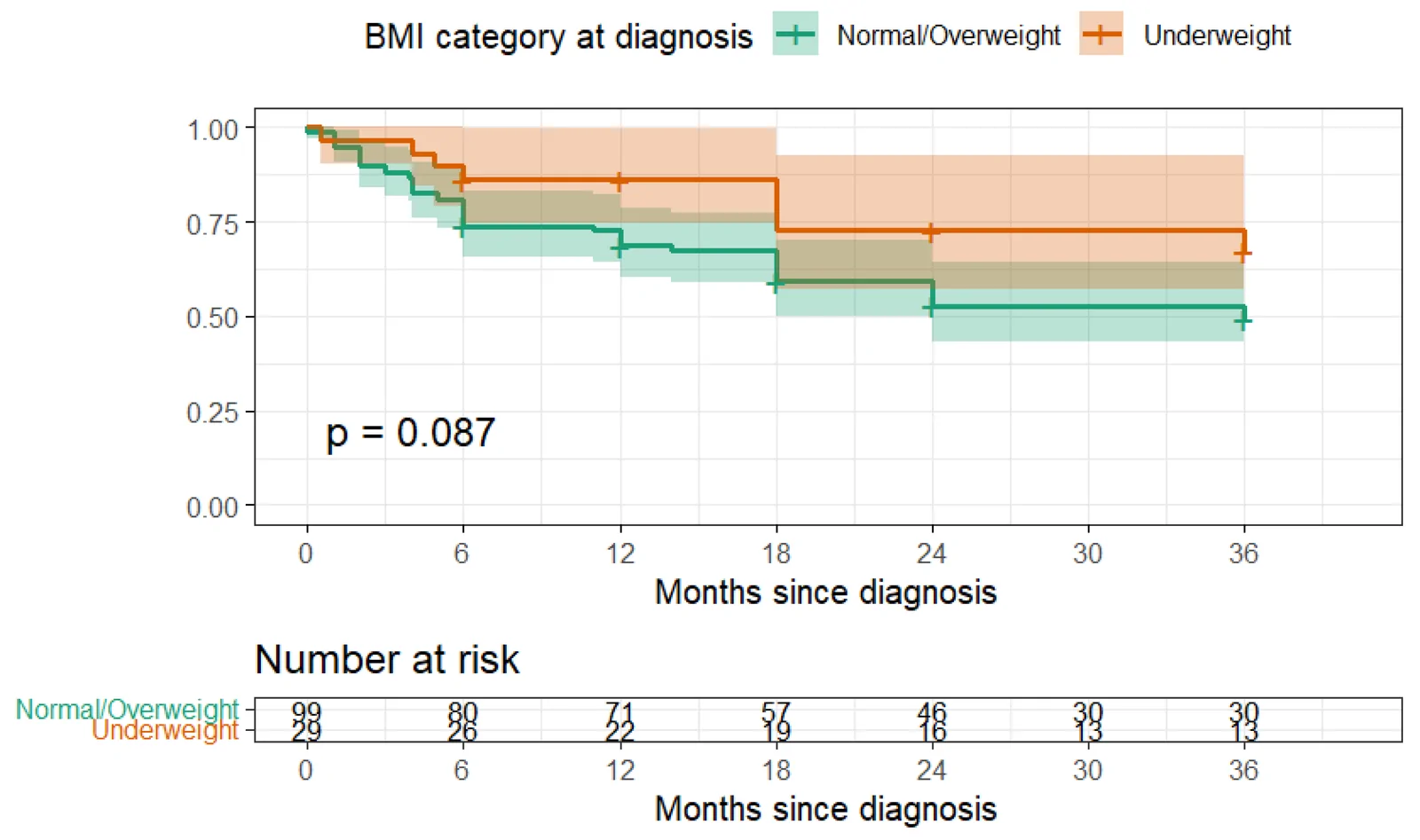
Kaplan–Meier estimates of relapse-free survival according to baseline nutritional status (underweight vs. non-underweight). Shaded areas represent 95% confidence intervals.

The results of the adjusted analysis are summarised in Figure 2 and Table 4.

## 4. Discussion

This multicentre paediatric cohort study evaluated nutritional status and linear growth from diagnosis through follow-up and investigated their relationship with treatment and subsequent disease course in children with IBD.

At diagnosis, approximately 25% of the paediatric cohort was classified as underweight, underscoring the substantial epidemiological burden of nutritional impairment in paediatric inflammatory bowel disease. This prevalence aligns closely with established historical data, which document malnutrition rates ranging between 60% and 80% in paediatric CD and 20% to 40% in UC cohorts [12]. The higher proportion of underweight patients in CD compared with in UC is in line with the known impact of small bowel involvement and transmural inflammation on nutrient absorption and growth [13,14,19,20]. No significant sex-related differences were observed, suggesting that malnutrition affects both males and females similarly.

An additional finding emerged when linear growth was evaluated alongside BMI. At diagnosis, stunting, defined as a height-for-age z-score (HAZ) ≤ −2 SD, was present in 7% of children, which is substantially less frequently than underweight status, affecting 23% of the cohort. These findings suggest that nutritional impairment at diagnosis was more frequently reflected by low BMI than by established linear growth impairment [21]. At 36 months, stunting was observed in 5.3% of patients with available anthropometric data, consistent with previous paediatric CD studies reporting growth failure in approximately 8% of patients at diagnosis and 5% during follow-up [22]. Similarly, a recent prospective multicentre study by Shimizu et al. reported growth impairment at diagnosis in 8.3% of children with CD and 6.2% of those with UC [23]. Previous studies have also shown that impaired growth velocity is closely associated with persistent inflammatory activity, further supporting the relationship between disease control and linear growth in paediatric IBD [22].

Given the close relationship between pubertal development and linear growth, pubertal status should also be considered when interpreting growth trajectories in paediatric IBD. In our cohort, pubertal status was available for 56% of patients, with a similar distribution between CD and UC. Recent evidence further highlights the complex interplay between disease activity, treatment, linear growth, and pubertal development in children with IBD [24].

However, both BMI and HAZ provide only a partial characterisation of nutritional status. BMI does not distinguish lean from fat mass, while HAZ reflects attained linear growth but may not fully capture recent changes in growth velocity. Importantly, alterations in body composition, particularly reduced lean and skeletal muscle mass, may occur even in children with preserved BMI [13,25]. In paediatric IBD, chronic inflammation, inadequate nutrient intake, increased metabolic demands, and reduced physical activity may contribute to alterations in body composition before they become apparent through conventional anthropometric measures. Evidence [26] also suggests that reduced muscle mass and sarcopenia may be associated with disease severity and poorer clinical outcomes, although their prognostic role in paediatric IBD remains to be fully established [5,12,27,28].

Beyond anthropometric measures, recent evidence suggests that dietary quality and the inflammatory potential of the diet may represent additional dimensions of nutritional assessment in children with Crohn’s disease [29]. More broadly, increasing evidence supports the role of dietary patterns and nutritional interventions as relevant components of IBD management [30].

The mean age at diagnosis was consistent with previous paediatric cohorts, in which most cases are diagnosed during adolescence [1]. Diagnostic delay did not differ between BMI groups, suggesting that malnutrition is more likely related to disease activity and inflammatory burden rather than delayed diagnosis [31]. Similarly, trends toward a higher prevalence of underweight in patients with ileal CD and extensive UC were observed, although not statistically significant, supporting the role of disease location and extent in nutritional impairment [32].

Laboratory findings further support the relationship between underweight status and systemic inflammatory burden. Elevated erythrocyte sedimentation rate and thrombocytosis were more frequent among underweight patients, consistent with previous evidence linking nutritional impairment with greater inflammatory activity in paediatric IBD. Other inflammatory and haematological markers, including C-reactive protein and haemoglobin, did not differ significantly according to nutritional status [33,34]. Faecal calprotectin was elevated in most patients regardless of BMI, suggesting widespread intestinal inflammation at diagnosis [35]. Ferritin levels were numerically higher in underweight patients, although the difference did not reach statistical significance. As ferritin is an acute-phase reactant, this finding may reflect the greater systemic inflammatory burden observed in underweight patients rather than differences in iron stores alone. In this context, inflammation-independent markers such as soluble transferrin receptor may provide a more reliable assessment of iron deficiency in patients with active IBD [36]. Overall, these findings support a close interplay between inflammation and nutritional status in paediatric IBD.

Regarding treatment, the proportion of patients receiving biologic therapy was numerically higher among underweight patients, although this difference did not reach statistical significance (*p* = 0.06). This finding may reflect a trend toward a more severe clinical presentation requiring early therapeutic escalation; however, it should be interpreted with caution [37,38,39]. EEN was widely used in CD, consistent with current guidelines recommending its use as first-line induction therapy [40,41,42]. The lack of association between EEN and BMI categories likely reflects its standardised use rather than differential clinical indication.

The majority of patients achieved normal weight during follow-up. However, a subset of patients remained underweight, highlighting the persistent and multifactorial nature of malnutrition in paediatric IBD. Conversely, some patients became overweight, underscoring the need for balanced and individualised nutritional management [43]. In parallel, the prevalence of stunting decreased modestly over time, particularly in patients with CD, while remaining largely stable in those with UC. These findings are consistent with previous reports showing that linear growth impairment is more common in Crohn’s disease than in ulcerative colitis and that it tends to improve with effective control of intestinal inflammation [44].

Relapse events were frequent, consistent with the relapsing–remitting nature of IBD [45,46]. Patients who were underweight at diagnosis did not have a significantly higher risk of subsequent relapse. Relapse-free survival did not significantly differ according to baseline nutritional status, and multivariable analysis confirmed that underweight status at diagnosis was not independently associated with relapse risk. Taken together, these findings suggest that baseline underweight status may represent a marker of inflammatory burden at diagnosis rather than an independent predictor of future disease relapse.

This study has several limitations. Its retrospective design may have introduced selection bias and incomplete data collection and precluded an a priori sample size calculation. The relatively small sample size, particularly in subgroup analyses, may have limited the statistical power. Nutritional assessment relied primarily on conventional anthropometric measures without direct assessment of body composition, while micronutrient and dietary data were not systematically collected. Pubertal status was available for only 56% of the cohort, limiting the interpretation of longitudinal growth trajectories. Potential inter-centre therapeutic heterogeneity, referral bias related to the tertiary-care setting, and the lack of standardised quality of life and treatment-adherence measures should also be acknowledged. Finally, despite multivariable adjustment, the retrospective observational design precludes causal inference. Nevertheless, this well-characterised multicentre cohort provides longitudinal data on nutritional status and linear growth, offering clinically relevant insights into their evolution and their relationship with disease course in paediatric IBD.

## 5. Conclusions

Malnutrition remains highly prevalent at diagnosis in paediatric IBD and is closely associated with inflammatory burden. Although most patients show improvement in nutritional status over time, a subset remains nutritionally compromised. These findings highlight the importance of the early and longitudinal assessment of nutritional status and linear growth as core components of paediatric IBD management. Further prospective studies with larger cohorts and more comprehensive nutritional assessment are needed to determine whether baseline nutritional status may serve as a prognostic marker and to guide personalised therapeutic strategies.

## 6. Future Perspectives

Future research should focus on prospective, adequately powered multicentre studies incorporating standardised assessments of nutritional status and body composition, including bioelectrical impedance analysis, dual-energy X-ray absorptiometry, and muscle ultrasound, together with systematic evaluation of micronutrient status and dietary intake. Longitudinal assessment of growth, body composition, nutritional and inflammatory biomarkers, and energy requirements should further clarify their relationship with treatment response and disease course. Integrating these parameters into prognostic models may facilitate the development of more personalised management strategies in paediatric IBD.

## Figures and Tables

**Table 1:** Baseline characteristics according to BMI category.

Variable	Total	Underweight	Normal/Overweight	*p*-Value
***Mean age at diagnosis, months***	157.2	163.1	155.5	0.22
*Sex, n (%)*				
Male	75 (59%)	20 (69%)	55 (56%)	0.20
Female	53 (41%)	8 (31%)	44 (44%)	
***Disease type, n (%)***				
Crohn’s disease (CD)	69 (54%)	21 (30%)	48 (70%)	0.022 *
Ulcerative colitis (UC)	59 (46%)	8 (15%)	50 (85%)	
***CD location (Paris), *** ***n (%)***				
Ileal involvement	40 (58%)	11 (28%)	29 (72%)	0.53
Non-ileal	29 (42%)	10 (34%)	19 (66%)	
***UC extent (Paris), *** ***n (%)***				
E1–E3	24 (41%)	3 (13%)	21 (87%)	0.60
E4 (pancolitis)	35 (59%)	6 (17%)	29 (83%)	
***CD behaviour *** ***at diagnosis (Paris)***				
B1	52 (75%)	11 (21%)	41 (79%)	0.003 *
B2-B3	17 (25%)	10 (59%)	7 (41%)	
***UC behaviour *** ***at diagnosis (Paris)***				
S0	33 (56%)	6 (18%)	27 (82%)	0.2
S1	26 (44%)	2 (8%)	24 (92%)	
***PCDAI at diagnosis (CD), mean (SD)***	27.7	27.8	27.6	0.90
	(17.7)	(17.4)	(15)	
***PUCAI at diagnosis (UC), mean (SD)***	39.8	25	42.3	0.1
	(24.2)	(20.2)	(24)	
***Diagnostic delay, months, median (range)***	4.5	4	4	0.17
	(0–38)	(0–21)	(0–38)	

Percentages were calculated based on available data; missing values were not included. * statistically significant. BMI, body mass index; CD, Crohn’s disease; PCDAI, Paediatric Crohn’s Disease Activity Index; PUCAI, Paediatric Ulcerative Colitis Activity Index; UC, ulcerative colitis.

**Table 2:** Laboratory and faecal parameters according to BMI category at diagnosis.

Variable	Cut-Off	Underweight (*n* = 29), *n* (%)	Normal/ Overweight (*n* = 99), *n* (%)	*p*-Value
*ESR*	>20 mm/h	24 (83%)	63 (64%)	0.02 *
*CRP*	>2 mg/L	13 (45%)	27 (27%)	0.15
*Haemoglobin*	<10 g/dL	10 (34%)	25 (25%)	0.26
*Platelets*	>350,000/µL	21 (72%)	53 (54%)	0.03 *
*Albumin*	<3.5 g/dL	7 (24%)	14 (14%)	0.37
*Ferritin*	<10 µg/L	5 (17%)	25 (25%)	0.32
*Faecal calprotectin*	>250 µg/g	24 (83%)	81 (82%)	0.90
	>500 µg/g	22 (76%)	74 (75%)	

Percentages were calculated based on available data; missing values were not included. * statistically significant. CRP, C-reactive protein; ESR, erythrocyte sedimentation rate.

**Table 3:** Prevalence of stunting (HAZ ≤ 2 SD) from diagnosis to 36-month follow-up, overall and according to disease type.

	Diagnosis	12 Months	24 Months	36 Months
*Overall, % stunted*	7.0%	5.9%	5.5%	5.3%
*CD, % stunted*	10.1%	7.7%	7.8%	6.7%
*UC, % stunted*	3.4%	3.7%	2.5%	3.3%
*Children with available HAZ data*	128	119	91	75

Percentages were calculated based on available data; missing values were not included. CD, Crohn’s disease; UC, ulcerative colitis.

**Table 4:** Logistic and Cox regression analyses of baseline underweight status and subsequent disease relapse.

Group	*n*	Univariate OR (95% CI, *p*)	Adjusted OR (95% CI, *p*)	Log-Rank *p*	Adjusted HR (95% CI, *p*)
*Pooled (CD + UC)*	128	0.44 (0.18–1.09, *p* = 0.075)	0.52 (0.2–1.33, *p* = 0.17)	0.081	0.59 (0.27–1.28, *p* = 0.18)
*CD*	69	0.48 (0.15–1.52, *p* = 0.21)	0.46 (0.14–1.56, *p* = 0.22)	0.320	0.64 (0.23–1.76, *p* = 0.38)
*UC*	59	0.53 (0.12–2.47, *p* = 0.42)	0.52 (0.11–2.46, *p* = 0.41)	0.316	0.54 (0.16–1.80, *p* = 0.32)

Adjusted for disease type, age at diagnosis, sex, biologic use at induction, pooled model additionally adjusted for disease type.

## Data Availability

The data generated and analysed in the current study are available from the corresponding author on reasonable request, subject to institutional approval due to ethical reasons.

## Abbreviations

The following abbreviations are used in this manuscript:

BMI	body mass index
CD	Crohn’s disease
CRP	C-reactive protein
EEN	exclusive enteral nutrition
ESR	erythrocyte sedimentation rate
IBD	inflammatory bowel disease
IBD-U	inflammatory bowel disease-unclassified
PCDAI	Paediatric Crohn’s Disease Activity Index
PUCAI	Paediatric Ulcerative Colitis Activity Index
UC	ulcerative colitis
VEO-IBD	very early-onset inflammatory bowel disease
